# Manganese neurotoxicity: new perspectives from behavioral, neuroimaging, and neuropathological studies in humans and non-human primates

**DOI:** 10.3389/fnagi.2013.00023

**Published:** 2013-06-24

**Authors:** Tomás R. Guilarte

**Affiliations:** Department of Environmental Health Sciences, Mailman School of Public Health, Columbia UniversityNew York, NY, USA

**Keywords:** manganese, neurotoxicity, Parkinson's disease, dopamine, motor function, cognitive function, working memory

## Abstract

Manganese (Mn) is an essential metal and has important physiological functions for human health. However, exposure to excess levels of Mn in occupational settings or from environmental sources has been associated with a neurological syndrome comprising cognitive deficits, neuropsychological abnormalities and parkinsonism. Historically, studies on the effects of Mn in humans and experimental animals have been concerned with effects on the basal ganglia and the dopaminergic system as it relates to movement abnormalities. However, emerging studies are beginning to provide significant evidence of Mn effects on cortical structures and cognitive function at lower levels than previously recognized. This review advances new knowledge of putative mechanisms by which exposure to excess levels of Mn alters neurobiological systems and produces neurological deficits not only in the basal ganglia but also in the cerebral cortex. The emerging evidence suggests that working memory is significantly affected by chronic Mn exposure and this may be mediated by alterations in brain structures associated with the working memory network including the caudate nucleus in the striatum, frontal cortex and parietal cortex. Dysregulation of the dopaminergic system may play an important role in both the movement abnormalities as well as the neuropsychiatric and cognitive function deficits that have been described in humans and non-human primates exposed to Mn.

## Introduction

Manganese (Mn) is an essential trace metal that is required for a number of enzymes important for normal cellular functions (Aschner and Aschner, 2005). However, excess accumulation of Mn in the brain results in a neurological syndrome with cognitive, psychiatric and motor abnormalities (Pal et al., 1999; Olanow, 2004; Perl and Olanow, 2007; Guilarte, 2010). Following excess exposure to Mn, the highest concentrations of Mn in the brain occur in the basal ganglia, specifically in the globus pallidus, caudate/putamen, and substantia nigra (Dorman et al., 2006; Guilarte et al., 2006a). These same studies have shown that Mn also accumulates in other brain structures within the cerebral cortex and in white matter (Dorman et al., 2006; Guilarte et al., 2006a). The accumulation of Mn in the basal ganglia is likely to be responsible for a form of parkinsonism with overlapping, but distinct clinical features with those seen in idiopathic Parkinson's disease (PD) (see below). Recently, there has been a great deal of debate in the scientific literature regarding the possibility that Mn may have an etiological role in idiopathic PD or accelerate the expression of PD (Racette et al., 2001, 2005). From a different perspective during the last decade there is mounting experimental evidence that exposure to Mn, at lower doses than those needed to produce motor function deficits, has a significant effect on executive function and cognition (Klos et al., 2006; Schneider et al., 2006, 2009; Roels et al., 2013). In this review, I examine the available evidence from human and non-human primate studies on the impact of elevated Mn exposures and its effects on motor function and cognitive domains.

## Manganese-induced parkinsonism

The first description of Mn-induced parkinsonism goes back to 1837 when Couper provided the sequelae of workers employed in the grinding of Mn oxide ore (Couper, 1837). In more modern times, there has been a number of reports describing clinical expression of parkinsonism in occupationally exposed workers (Mena et al., 1967; Cook et al., 1974; Huang, 2007; also see studies in Perl and Olanow, 2007 and in Guilarte, 2010) with clear evidence that excess exposures to Mn produces motor function deficits in humans and non-human primates that resemble some aspects to those expressed in idiopathic PD (Perl and Olanow, 2007; Guilarte, 2010) as well as more subtle effects on motor function, specifically fine motor control depending upon the level of exposure (Perl and Olanow, 2007; Guilarte, 2010). However, there are clear differences between Mn-induced parkinsonism and idiopathic PD from a clinical perspective and in the underlying neuropathology (Perl and Olanow, 2007; Guilarte, 2010) (see next section).

The most compelling human evidence of Mn-induced parkinsonism in the last decade comes from a very unfortunate human experiment in which young drug users inject very high levels of Mn from use of home-made psychostimulant preparations (ephedron, also called methcathinone) (de Bie et al., 2007; Meral et al., 2007; Sanotsky et al., 2007; Sikk et al., 2007, 2010, 2013; Selikhova et al., 2008; Stepens et al., 2008, 2010; Varlibas et al., 2008; Colosimo and Guidi, 2009; Yildirim et al., 2009; Iqbal et al., 2012). These cases of young drug users with clinical parkinsonism as a result of drug abuse are reminiscent of young addicts injecting 1-methyl-4-phenyl-1,2,3,6-tetrahydropyridine (MPTP) and expressing clinical parkinsonism in the early 1980s (Langston et al., 1983). The ephedron home-made preparations is the result of using potassium permanganate to oxidize ephedrine or pseudoephedrine and it is injected with minimal purification; thus, users inject very high doses of Mn. These ephedron users exhibited clinical parkinsonism that is not responsive to L-dopa therapy (Sanotsky et al., 2007; Selikhova et al., 2008; Stepens et al., 2008; Colosimo and Guidi, 2009; Sikk et al., 2013). This clinical observation suggests that the underlying neurobiology associated with Mn-induced parkinsonism is different from the well-recognized loss of dopamine neurons in the substantia nigra pars compacta (SNpc) that is responsive to L-dopa therapy in idiopathic PD patients (Savitt et al., 2006) and in MPTP subjects (Forno et al., 1993; Forno, 1996) and MPTP exposed non-human primates (Nerastet et al., 1994).

The etiological role of Mn in producing the motor function deficits in these relatively young ephedron users can be confirmed by the extremely high levels of Mn measured in their blood (Selikhova et al., 2008; Stepens et al., 2008; Sikk et al., 2010, 2013) and the bilateral hyperintensive signal in the basal ganglia observed in T1-weighted magnetic resonance imaging (MRI) consistent with excess accumulation of Mn in the brain (Selikhova et al., 2008; Stepens et al., 2008; Sikk et al., 2010, 2013). Importantly, in Eastern European countries in which there is expression of Mn-induced parkinsonism in ephedron drug users, the synthesis of the ephedron uses potassium permanganate to oxidize ephedrin or pseudoephedrin. On the other hand, in the United States drug users make the same ephedrone preparation, however, they oxidize the ephedrine with chromate and there is no evidence of parkinsonism (Stepens et al., 2008). This provides compelling evidence that the culprit in the home-made ephedron preparations used in Eastern European countries is the high levels of Mn that are injected by these individuals.

## Manganese-induced parkinsonism: degeneration or dysfunction of dopaminergic neurons?

### Human studies

In the last decade there has been a great deal of debate in the scientific literature about the potential role of Mn on the etiology of idiopathic PD. Epidemiological studies indicate that long-term exposure (>20 years) to Mn is associated with idiopathic PD (Gorell et al., 1999). Studies in welders have suggested that Mn exposure precipitates an earlier expression of idiopathic PD (Racette et al., 2001, 2005). However, the studies in welders have been criticized from several perspectives (see Ravina et al., 2001; Guilarte, 2010) and a confounding problem in human studies is that workers occupationally exposed to Mn could have an underlying susceptibility to develop PD. Thus, it is difficult to know whether the Mn exposure is the etiological agent that induces idiopathic PD or whether there is a coincidental Mn exposure in individuals that are destined to express the disease. In an effort to examine the potential role of Mn in idiopathic PD, a recent study used [^18^F]-Fluoro-L-Dopa Positron Emission Tomography ([^18^F]-FDOPA PET) imaging on 20 asymptomatic welders (exposed to welding fumes containing Mn), 20 subjects with idiopathic PD and 20 normal controls (Criswell et al., 2011). [^18^F]-FDOPA PET is a non-invasive neuroimaging method to assess presynaptic dopamine terminal activity *in vivo* and has been used in idiopathic PD patients as a marker of dopamine terminal integrity (Gallagher et al., 2011; Jaimini et al., 2013). Notably, [^18^F]-FDOPA uptake is dramatically decreased with a distinct regional pattern in the caudate and putamen of idiopathic PD patients (Morrish et al., 1996; Nurmi et al., 2001; Hilker et al., 2005; Gallagher et al., 2011). An important aspect of the investigation by Criswell and colleagues is that welders were relatively young (mean age 45.2 years), apparently asymptomatic and in good health, thus reducing the possibility of expressing an underlying idiopathic PD etiology in order to minimize the likelihood of coincidental Mn exposure with idiopathic PD. The authors found that welders expressed significantly elevated levels of blood Mn and a higher pallidal index (the pallidal index is a measure of Mn accumulation on the globus pallidus as a ratio of the signal intensity in the globus pallidus over the intensity in the frontal white matter using T1-weighted MRI) than controls and subjects with idiopathic PD; thus confirming that the welders were actively exposed to Mn. Upon neurological examination, welders demonstrated a slightly elevated average United Parkinson's Disease Rating Scale-subscale 3 (UPDRS3) score relative to controls indicative of subtle effects of Mn on motor function while the idiopathic PD subjects had a much higher score consistent with their diagnosis. The results of the [^18^F]-FDOPA-PET studies indicated that the welders had a small (10%) but significantly lower level of [^18^F]-FDOPA uptake in the caudate nucleus relative to controls but no effect on the anterior or posterior putamen (Criswell et al., 2011). On the other hand, idiopathic PD patients expressed the expected pattern of [^18^F]-FDOPA uptake deficits in the caudate and putamen relative to controls. That is, idiopathic PD subjects had marked reductions in [^18^F]-FDOPA uptake in the putamen (~52% in the posterior putamen and 35% in the anterior putamen) with a smaller reduction in the caudate nucleus (~17%) (Criswell et al., 2011). This study showed that the pattern of the impairment in dopamine terminal function in welders actively exposed to Mn-containing welding fumes is not the same to that observed in idiopathic PD. It should also be noted that the interpretation of the decrease in [^18^F]-FDOPA uptake in the caudate in the welders exposed to Mn should not necessarily be interpreted as representative of dopamine terminal degeneration as is the case in idiopathic PD. It is possible that Mn exposure could alter enzymes that are responsible for [^18^F]-FDOPA metabolism and this possibility needs to be ruled out. Other types of PET studies should also be performed that would be more representative of dopamine terminal integrity and are less likely to be influenced by changes in dopamine metabolizing enzymes and/or changes in dopamine levels. For example, [^11^C]-dihydrotetrabenazine (DTBZ) PET for vesicular monoamine trasnporter type-2 (VMAT-2) is more likely to represent structural changes in dopamine terminals than [^18^F]-FDOPA PET when it relates to studies with Mn.

The findings of Criswell et al. (2011) do suggests that the small but significant Mn-induced decrease in [^18^F]-FDOPA uptake in the caudate nucleus may be associated with potential effects on cognitive domains since the caudate nucleus has extensive connections to cortical structures, especially to frontal cortical areas that are involved in executive function (see below). Consistent with this hypothesis, several studies in early idiopathic PD patients show that reductions in [^18^F]-FDOPA uptake in the caudate nucleus are associated with deficits in working memory performance and executive function (Rinne et al., 2000; Jokinen et al., 2009, 2013), effects that were not associated with reduction in [^18^F]-FDOPA uptake in the putamen.

This recent study using state-of-the-art PET instrumentation and analysis provides evidence of a relative lack of dopamine neuron terminal degeneration in welders expressing small increases on the UPDRS3 scale. Previous studies in smelter workers with clinical parkinsonism have reported normal [^18^F]-FDOPA-PET in the striatum (Huang, 2007). Further, neuroimaging studies performed in the ephedrone users indicating normal levels of dopamine terminals, based on dopamine transporter (DAT) levels, in the striatum using SPECT imaging despite the fact that they express clinical parkinsonism (Selikhova et al., 2008; Colosimo and Guidi, 2009; Sikk et al., 2010, 2013; Iqbal et al., 2012). Thus, the most recent human studies with state-of-the-art neuroimaging methodologies indicate that there is a relative lack of dopamine neuron terminal degeneration in the caudate and putamen as a result of Mn exposure. These findings raise the important question, what is the underlying neurobiological deficit in dopaminergic neurons in Mn-induced parkinsonism?

### Non-human primate studies

During the last decade, our laboratory in collaboration with a multidisciplinary group of investigators has been studying the neurological consequences of chronic exposures to moderate levels of Mn (Guilarte et al., 2006a,b, 2008a,b; Burton and Guilarte, 2009; Burton et al., 2009; Verina et al., 2011, 2013; Schneider et al., 2006, 2009). These on-going studies use research naïve *Cynamolgos macaques* (5–6 years of age at the initiation of the study) in which there is extensive behavioral and neuroimaging assessment prior to (baseline) and at two different time points after initiation of Mn administration (Guilarte et al., 2006b, 2008a). After the animals have gone through the behavioral and neuroimaging protocols [the latter includes T1-weighted MRI (MRI), Magnetic Resonance Spectroscopy (MRS), PET and currently Diffusion Tensor Imaging (DTI)] *ex vivo* neurochemical and neuropathological confirmation of the PET findings as well as other neurochemical and neuropathological outcomes are performed. One of the neuroimaging studies performed is to assess DAT levels as a putative synaptic marker of dopamine terminal integrity in the caudate and putamen using [^11^C]-methylphenidate PET. Another PET study uses a continuous infusion of [^11^C]-raclopride (a D2-dopamine receptor ligand) with amphetamine challenge in order to measure both D2-dopamine receptor (D2R) levels and *in vivo* dopamine release (Laruelle, 2000; Zhou et al., 2006). Importantly, the imaging studies provide an internal control since each animal receives a “baseline” (prior to Mn exposure) imaging set (MRI/MRS/DTI/PET). In addition, to the Mn-exposed animals, an “imaged-control” group was used. This group of animals goes through the same imaging protocol, but they do not receive Mn. A second “naïve controls” group was also used for the neuropathological endpoints and this group of animals does not receive Mn exposure nor does it go through the imaging protocol.

The results of our PET studies demonstrate that chronic exposure to moderate levels of Mn does not produce the loss of dopamine terminals, i.e., there was a lack of dopamine terminal degeneration in the caudate and putamen based on [^11^C]-methylphenidate PET for DAT under our experimental Mn dose and exposure conditions (Guilarte et al., 2006b, 2008a). On the other hand, we found a highly significant effect of Mn on dopamine terminal dysfunction since there was a marked (~60% from baseline) and progressive decrease of *in vivo* dopamine release in the striatum of Mn-exposed animals measured by PET (Guilarte et al., 2006b, 2008a). This effect was not observed in the “imaged-control” group. Therefore, the impairment of *in vivo* dopamine release was the direct result of the Mn administration (Guilarte et al., 2008a).

One potential explanation for the impairment of *in vivo* dopamine release measured by PET in the Mn-exposed animals is that Mn produces a decrease in the synthesis of dopamine, thus resulting in lower levels of synaptic (vesicular) dopamine available for release. To answer this questions, a number of *ex vivo* neurochemical studies were performed in the caudate and putamen of the same animals in which PET studies were performed. The results show that when all control groups were combined (imaged-controls and naïve controls) and used as a referent group, there were no significant differences on the levels of dopamine and metabolites in the caudate and there was only an effect of Mn on dopamine levels in the putamen when compared to the naïve controls only (Guilarte et al., 2008a). A similar effect was observed for DAT and vesicular dopamine transporter-2 (VMAT-2) in the caudate and putamen. Lastly, there was no effect of Mn-exposure on DAT or tyrosine hydroxylase (TH) immunostaining in the caudate and putamen. In summary, the non-human primate studies performed under highly controlled experimental and Mn dosing conditions indicate that exposure to moderate levels of Mn does not result in dopamine neuron degeneration as in idiopathic PD but it produces significant dopamine neuron dysfunction. We have proposed that the subtle fine motor control deficits observed in these animals is the result of a dopamine release deficit (Guilarte et al., 2006b, 2008a; Guilarte, 2010). Our non-human primate findings are consistent with the most recent neuroimaging studies in humans indicating a lack of dopamine neuron terminal degeneration in subjects with clinical parkinsonism resulting from ephedrone use (Selikhova et al., 2008; Colosimo and Guidi, 2009; Sikk et al., 2010; Iqbal et al., 2012).

While the current review does not include rodent studies, there is recent evidence in the literature that rodents exposed to Mn also have impairment in dopamine release with no change in total tissue dopamine levels, dopamine neuron terminals in the striatum, or TH-positive dopaminergic cell bodies in the SNpc (Vidal et al., 2005; Peneder et al., 2011). Combined these studies provide evidence that Mn-induced parkinsonism may be the result of the inability of dopamine neuron terminals to release dopamine rather than a decrease of dopamine synthesis in intact terminals and/or the loss of dopamine as a result of terminal degeneration. These findings provide a logical explanation to the evidence that Mn-induced parkinsonism is not responsive to L-dopa therapy (Lu et al., 1994; Sanotsky et al., 2007; Selikhova et al., 2008; Stepens et al., 2008; Colosimo and Guidi, 2009; Sikk et al., 2013) as is idiopathic PD since in Mn-induced parkinsonism there is no apparent loss of dopamine terminal or dopamine levels in the striatum. Our findings in non-human primates that Mn impairs dopamine release needs to be confirmed in humans exposed to Mn. Collectively, our PET findings implicate a novel mechanism by which dopamine neuron dysfunction, that is, the inability to release dopamine, rather than a degenerative process can result in clinical parkinsonism as a result of Mn exposure.

## Effects of manganese exposure on neuropsychiatric symptoms and cognitive function

The clinical expression of Mn-induced neurotoxicity in humans has been described as a continuum with different stages with distinct clinical manifestations (Mergler et al., 1999). Humans exposed to Mn express changes in sleep patterns and mood with uncontrollable laughter and crying, euphoria, aggressiveness, hallucinations and psychosis (Donaldson, 1987). An acute effect of Mn intoxication has been described as a clinical condition with symptoms reminiscent of schizophrenia and amphetamine-induced psychosis (Donaldson, 1987; Perl and Olanow, 2007). Although the current knowledge on the psychiatric aspects of chronic Mn exposure are limited, recent studies indicate that humans with increased exposure to Mn (Bowler et al., 2003, 2006, 2007a,b; Josephs et al., 2005; Park et al., 2009) and from medical conditions that results in increased Mn accumulation in the brain (Mirowitz et al., 1991; Klos et al., 2006) express impairments in attention and learning and memory function suggestive of frontal lobe and subcortical dysfunction. Studies have shown that workers occupationally exposed to Mn have a higher incidence of neuropsychiatric symptoms than referents (Bouchard et al., 2007) and elevated levels of Mn markedly increase neuropsychiatric symptoms associated with alcohol abuse (Sassine et al., 2002). An increasing number of reports also indicate effects on working memory (Bowler et al., 2003, 2006, 2007a,b; Klos et al., 2006) and poor cognitive performance (Mergler and Baldwin, 1997; Santos-Burgoa et al., 2001; Bowler et al., 2003, 2007a,b; Klos et al., 2006). Importantly, the effects of Mn on working memory points to deficits in frontal lobe function, a brain region known to be involved in neuropsychiatric illnesses such as schizophrenia (Goldman-Rakic, 1999; Abi-Dargham et al., 2002). A growing number of reports in children with elevated exposures to Mn indicate below average performance in verbal and visual memory tests (Woolf et al., 2002; Wright et al., 2006) and intellectual function (Wasserman et al., 2006; Claus Henn et al., 2010; Bouchard et al., 2011; Menezes-Filho et al., 2011; Khan et al., 2012). Children followed from birth through the early years have cord blood Mn concentrations that were negatively correlated with scores on attention, non-verbal memory and hand skills (Takser et al., 2003). Despite these studies, basic knowledge on mechanism(s) by which Mn produces psychiatric symptoms and cognitive impairment is lacking. Therefore, a great deal can be learned not only from Mn effects on basal ganglia function but also from effects on cognitive domains associated with the frontal cortex and other cortical and subcortical structures.

## The cerebral cortex—a novel target of manganese neurotoxicity

There is a paucity of knowledge on the neuropathological consequences of excess Mn accumulation in cortical regions and specifically in the frontal cortex. This is based in part on the fact that: (1) most studies on Mn-induced neurochemical and neuropathological changes have been focused on basal ganglia structures due to its association with movement abnormalities and parkinsonism, and (2) Mn accumulates to a high degree in the basal ganglia. Besides the suggestion from neuropsychological and cognitive tests of frontal cortex involvement in Mn-induced neurological dysfunction, a review of the literature brings to light a lack of neuropathological studies in which Mn effects on the cerebral cortex have been performed. It is only recently when neuroimaging studies have interrogated cortical regions to examine their susceptibility to Mn-induced neurotoxicity. In this context, our recent studies in non-human primates have reported proton MRS metabolite changes in Mn-exposed animals (Guilarte et al., 2006a). This includes a decrease in N-acetylaspartate (NAA) to creatine (Cr) ratio (NAA/Cr) in the parietal cortex with a nearly significant decrease (*p* = 0.055) in frontal white matter (Guilarte et al., 2006a). A decrease in the NAA/Cr ratio is representative of neuronal dysfunction and/or neuronal loss (Clark, 1998; Block et al., 2002). Since this original publication, two human studies have described effects of Mn on brain metabolites in the cerebral cortex. Chang et al. (2009) have shown that cognitive decline in welders was associated with a decrease in myoinositol/creatine (mI/tCr) ratio in the anterior cingulate cortex indicative of glial involvement. More recently, another MRS study in smelters showed a small but significant decrease in NAA/tCr ratio in the frontal cortex that was strongly correlated with cumulative Mn exposure (Dydak et al., 2011). Therefore, there is emerging evidence that exposure to Mn results in altered levels of brain metabolites in the cerebral cortex that reflect neuronal loss or dysfunction and glial cell activation. The only other evidence describing cortical involvement with brain Mn accumulation is a case report of an individual exhibiting progressive dementia, and extrapyramidal syndrome with an elevated Mn body burden (Banta and Markesbery, 1977). Brain biopsy and examination of cortical tissue revealed numerous neuritic plaques and neurofibrillary tangles in the right frontal lobe typical of Alzheimer's disease (AD) (Banta and Markesbery, 1977).

## Neuropathological changes in the frontal cortex of mn-exposed non-human primates

Previous reports from our on-going studies on the neurological effects of Mn in non-human primates have provided compelling evidence of Mn-induced pathology in the frontal cortex of young, research naïve animals (Guilarte et al., 2008b; Verina et al., 2013). Using microarray technology in frontal cortex tissue from Mn-exposed and control animals, we found significant alterations in genes with biological functions associated with: (1) cholesterol metabolism and transport, (2) axonal/vesicular transport, (3) inflammation and the immune response, (4) cell cycle regulation and DNA repair, (5) and proteasome function and protein folding and turnover. The most highly upregulated gene was β-amyloid precursor-like protein 1 (APLP1), a member of the amyloid precursor protein (APP) family associated with AD (Guilarte et al., 2008b). The increase in APLP1 gene expression was confirmed at the protein level using immunohistochemistry. We also found diffused β-amyloid plaques (6E10 antibody immunohistochemistry) in the frontal cortex from Mn-exposed animals that were not observed in age-matched controls. These findings were unexpected as these were young adolescent animals and normally non-human primates do not express β-amyloid diffuse plaques at an early age, although there is evidence of diffused β-amyloid plaques in aged (>20 years of age) non-diseased monkeys (Kimura et al., 2003, 2005). Examination of frontal lobe tissue also provided evidence of cortical and subjacent white matter degeneration based on silver staining. In the gray matter, histological staining provided evidence of neurons with a significant degree of intracytoplasmic vacuolization. In some of the animals, we observed neurons with hypertropic nuclei, a condition that has been associated with the early stages of AD (Iacono et al., 2008, 2009). Histological assessment of the frontal cortex also showed cells with apoptotic stigmata and astrocytosis in both the gray and white matter. More recently, we have reported evidence of α-synuclein aggregation in the frontal cortex gray and white matter from the same Mn-exposed animals (Verina et al., 2013). As noted earlier, these Mn-exposed animals expressed a near significant (*p* = 0.05) decrease in NAA/Cr ratio in the frontal cortex white matter (Guilarte et al., 2006a) consistent with the observation of white matter degeneration in post-mortem brain tissue. Therefore, our studies provided the first evidence of significant pathology in the frontal and parietal cortex of non-human primates exposed to Mn.

Recent human studies also support neurodegenerative changes resulting from Mn exposure in frontal cortex white matter. Stepens et al. (2010) report that individuals injecting ephedron-containing Mn express white matter abnormalities based on DTI. The authors describe evidence of diffuse white matter changes reflected by reductions in fractional anisotropy (FA) in the ephedron users. They also find effects specific to white matter underlying the right ventral premotor cortex and the medial prefrontal cortex. The authors indicate that the clinical features of these ephedron users point to a disorder of higher-level motor programming and that the pattern of motor function deficits resemble executive function deficits similar to those displayed by patients with prefrontal cortex lesions (Stepens et al., 2010). Another human study examining white matter ultrastructural integrity in welders also reveal white matter changes measured by DTI (Kim et al., 2011). They show that FA was significantly reduced in the corpus callosum and frontal white matter of welders. The FA values in these white matter regions was significantly associated with blood Mn levels and pallidal index. Importantly, the degree of FA disruption was associated with impaired attention, lower working memory and deficits in executive function tests (Kim et al., 2011).

These findings provided strong evidence that the frontal cortex gray matter and subjacent white matter are vulnerable, but previously unrecognized targets for Mn-induced neurotoxicity despite the fact that Mn accumulates in cortical structures at significantly lower concentrations than in the basal ganglia. These observations suggest that the neurotoxicological effects of Mn are not solely based on the degree to which Mn accumulates in different brain regions but they are also based on the vulnerability of a specific brain region to Mn-induced neurotoxicity. The emerging evidence in humans and non-human primates suggest that future studies on subjects with environmental and occupational exposures to Mn or in patients with medical conditions in which excess brain Mn accumulation occurs should be tested for neuropsychiatric symptoms and cognitive function deficits.

## Effects of manganese exposure on working memory

In the previous section evidence is provided that exposure to elevated levels of Mn results in detrimental effects on cortical structures, specifically the frontal and parietal cortex. Recent human and non-human primates studies suggest that a resulting effect of Mn-induced neuropathology in the frontal cortex is working memory deficits. Chang et al. (2010) report that welders with chronic Mn exposure express increased brain activity measured by functional MRI in working memory networks during the 2-back verbal working memory task. They interpret these findings as the welders requiring more neural resources in working memory networks to compensate for subtle deficits in working memory. In another study, Wasserman et al. (2011) found significant associations between Mn levels in drinking water and reductions in Perceptual Reasoning and Working Memory scores.

Our non-human primate studies were the first to provide initial evidence of Mn effects on working memory under highly controlled experimental conditions (Schneider et al., 2006, 2009). We showed that chronic Mn exposure resulted in deficits in non-spatial and spatial working memory. Non-spatial working memory assessed by delayed matching to sample performance appeared to be more affected than spatial working memory using a variable delayed response task (Schneider et al., 2009). In general, the human and non-human primate studies provide substantial evidence for impairments of cognitive domains that are mediated by the frontal cortex. Further, the non-human primate findings also implicate brain metabolite changes in the parietal cortex, a brain region that is important for working memory performance and plays an important role in integrating sensory information and visuo-spatial processing (Constantinidis and Wang, 2004; Seger, 2006; Linden, 2007).

## Can dopamine neuron dysfunction in the striatum and/or frontal cortex explain the working memory deficits observed in Mn exposed non-human primates?

Working memory is closely associated with frontal cortex function (Constantinidis and Wang, 2004; Linden, 2007) and dopamine neurotransmission in the striatum (Rinne et al., 2000; Sawamoto et al., 2008; Jokinen et al., 2009) and the frontal cortex (Brozoski et al., 1979; Rotaru et al., 2007). The dopamine cell bodies located in the SNpc project to the caudate and putamen and this nigrostriatal system is involved in motor control. In addition, there are direct mesolimbic dopaminergic projections from the ventral tegmental area to the frontal cortex (Bjorklund and Dunnett, 2007). The caudate nucleus receives dopaminergic input from the SNpc and it can influence frontal cortex function via well-defined frontostriatal circuits (Alexander et al., 1986; Seger, 2006). Human and non-human primates studies show that the dorsolateral prefrontal cortex (DLPFC) is an important region for the execution of working memory tasks with reciprocal connections to other cortical structures such as the parietal, temporal and cingulate cortex and these combined participate in a cortical network related to working memory (Kubota and Niki, 1971; Petrides et al., 1993; Berman et al., 1995; Cohen et al., 1997).

Lesions or dysfunction of the caudate nucleus has been reported to produce impairment in the delayed response tasks that assesses working memory (Levy et al., 1997; White, 2009). Relevant to our own studies, Mn-exposed animals have impairments of both spatial and non-spatial working memory (Schneider et al., 2009) and they also express a significant impairment of *in vivo* dopamine release in the striatum (Guilarte et al., 2008a). Further, welders exposed to Mn express an early deficit in dopamine neuron function specific to the caudate nucleus and not the putamen (Criswell et al., 2011). These findings suggest that dopamine neuron dysfunction via impairment of dopamine release in the striatum and specifically in the caudate may be associated with the working memory deficits expressed in Mn-exposed non-human primates and in humans. Other studies have shown that the levels of NAA in the DLPFC predict the activation of cortical regions involved in the execution of working memory tasks such as the frontal, parietal and temporal cortices and this network has been found to be affected in mental disorders such as schizophrenia (Bertolino et al., 2000; Castner et al., 2004). Postmortem studies in the frontal cortex of Mn-exposed non-human primates have found a significant degree of neuronal degeneration with diffused β-amyloid plaques and α-synuclein aggregation (Guilarte et al., 2008b; Verina et al., 2013) implicating a potentially important role of this neuropathology in the working memory deficits observed in Mn-exposed non-human primates (Schneider et al., 2009). Imaging studies in welders exposed to Mn support a Mn-induced neuronal cell death or dysfunction in the frontal cortex based on decreased NAA/tCr ratio (Dydak et al., 2011), an effect that was associated with cumulative Mn exposure. Combined these studies provide evidence that several brain regions (i.e., the caudate nucleus, the frontal cortex and the parietal cortex) within the working memory network appear to have substantial neuropathology and/or dysfunction as a result of chronic exposure to Mn.

Experimental animal and human studies have shown that dopamine is a key neurotransmitter in the regulation of working memory in the frontal cortex and caudate nucleus (Levy et al., 1997; Aalto et al., 2005; Cools et al., 2008; Landau et al., 2009; Backman et al., 2011; Cools and D'Esposito, 2011). Microdialysis studies have shown that working memory tasks induce the release of dopamine in the prefrontal cortex of monkeys (Watanabe et al., 1997) and rats (Phillips et al., 2004) and there is increased blood flow to prefrontal and parietal cortex in humans performing working memory tasks (Bertolino et al., 2000; Cabeza and Nyberg, 2000). Other studies have shown that D1-dopamine receptor (D1R) antagonists can impair working memory (Sawaguchi and Goldman-Rakic, 1991) while low doses of D1R agonists can improve working memory (Arnsten et al., 1994). Contrary to using low doses of dopamine receptor agonists, high doses of D1R agonists also impair working memory performance, an effect that is abrogated by pretreatment with a D1R antagonist (Zahrt et al., 1997; Goldman-Rakic et al., 2000). These findings suggest that either low levels or excessive levels of D1R dopamine receptor stimulation can have a negative impact on working memory performance (Goldman-Rakic et al., 2000; Cools and D'Esposito, 2011). Based on this literature, it is likely that the impairment of *in vivo* dopamine release measured in the striatum of Mn-exposed animals may be responsible for their impairment in working memory (see Guilarte et al., 2008a; Schneider et al., 2009). Alternatively, it is possible that chronic Mn exposure may also alter *in vivo* dopamine release in the frontal cortex, and along with deficits of dopamine release in the caudate nucleus may precipitate deficits on working memory performance.

## Analysis of *In vivo* dopamine release in the frontal cortex: PET imaging with [^11^C]-FLB 457

While the displacement of D2R specific PET ligands such as [^11^C]-raclopride by an acute amphetamine challenge has been validated and used extensively to measure *in vivo* dopamine release in the striatum (Laruelle, 2000). The use of this methodology is just emerging for the cerebral cortex (Narendran et al., 2009, 2011a,b, 2013). Since dopamine innervation to cortical structures is significantly lower than to the striatum, that is, dopamine terminals and dopamine receptor levels are much lower in the frontal cortex than in the caudate/putamen, *in vivo* dopamine release PET in cortical structures is a much more difficult task to perform. However, the development and use of high affinity D2R-PET ligands such as [^11^C]-FLB 457 (*K*d = 0.06 nM) and [^18^F]-fallypride (*K*d = 0.14 nM) have made such studies possible. Several publications have now described the reliability of using [^11^C]-FLB 457 to measure *in vivo* dopamine release in the cerebral cortex of humans and non-human primates (Narendran et al., 2009, 2011a,b, 2013). Further, a recent study has shown that the degree of [^11^C]-FLB 457 binding potential reduction measured by PET was directly associated with the amount of extracellular dopamine release induced by the acute amphetamine administration (Narendran et al., 2013). In summary, the ability to measure *in vivo* dopamine release in the cerebral cortex using PET is an extremely valuable approach to understand the molecular basis of the working memory impairments observed in humans and non-human primates exposed to Mn. We are currently performing these types of studies in our Mn-exposed animals in order to make associations between *in vivo* dopamine in cortical regions and working memory performance.

## Summary

In the last decade there has been significant progress using state-of-the-art neuroimaging and behavioral methodologies that have opened up a new understanding of Mn neurotoxicology. While historically the focus of Mn neurotoxicity has been associated with parkinsonism as a result of the high levels of exposure that occurred in the mining and processing of Mn ore and in other occupational settings, the last decade has brought about compelling experimental evidence that at lower cumulative doses of Mn that are likely to occur from occupational and environmental exposures, other non-motor neurological effects appear to be more prevalent and these seem to be associated with cognitive function deficits. The later may be the result of Mn producing brain chemistry and structural changes in cortical regions, and the frontal and parietal cortex appear to be sensitive targets. Lastly, because of its relevance to motor and cognitive domains, it is possible that dysfunction of the dopaminergic system could be a common mechanism by which Mn could have an impact on both cognitive and motor function deficits observed in humans and non-human primates.

### Conflict of interest statement

The author declares that the research was conducted in the absence of any commercial or financial relationships that could be construed as a potential conflict of interest.
